# Delivery of Retained Second Twin in Case of Omphalopagus Conjoined Twins: Abdominovaginal Approach

**DOI:** 10.1155/2018/9319721

**Published:** 2018-12-05

**Authors:** Wondimagegnehu Sisay Woldeyes

**Affiliations:** Assistant Professor of Obstetrics and Gynecology, Hawassa University, College of Medicine and Health Sciences, Ethiopia

## Abstract

Conjoined twin is a rare complication of monozygotic twins resulting from incomplete splitting of an embryo into two separate twins or early secondary fusion of two originally separated embryos. When diagnosed at early gestation, one can get adequate time to counsel the family on whether to continue with the pregnancy and proper intervention can be planned. On the other hand, undiagnosed cases may be first recognized in labor after they have caused labor-related complications. In the present case report, an undiagnosed case of a conjoined twin has presented in labor followed by delivery of one baby with the retention of the second baby. This presented a unique challenge to the managing team and required hysterotomy to effect delivery of the unborn baby. We report this case to highlight the importance of early diagnosis of a conjoined twin. On another hand where this does not happen and a conjoined twin is suspected for the first time after it has caused labor-related complications, the management should be individualized based on the clinical circumstance.

## 1. Introduction

Conjoined twins are one of the unique complications of monozygotic twins characterized by conjunction ranging from simple fusion of ectodermal structures to an extreme case where one of the twins is contained in the other. It is a rare event with an incidence ranging from 1 in 50,000 to 1 in 100,000 live births [1]. They result from aberrations in the twinning process in monozygotic twins. This can be due to incomplete splitting of an embryo into two separate twins or early secondary fusion of two originally separated embryos [2, 3].

Most of the evidence on diagnosis, management, and outcome of conjoined twins is based on case reports, case serious, and expert opinions. In this regard, there are ultrasound criteria for the diagnosis of conjoined twins at early gestation. With early diagnosis, time is enough for parents to decide whether to continue the pregnancy. Where discontinuation of the pregnancy is chosen, termination with the usual medical or surgical approaches can be carried out. On the other hand, if pregnancy continues by parents' choice or detected at advanced gestational age caesarean section it can be carried out with generous abdominal and uterine incision. On the other hand, in a situation where these cases are diagnosed intrapartum after they have developed labor abnormalities, it is unlikely that conjoined twin is considered as a cause and leads to labor ward tragedy and panic [4–8]. In the present case report, an undiagnosed case of a conjoined twin was presented in labor followed by delivery of one baby with the retention of the second baby. We managed this case in an individualized manner, with hysterotomy for manipulation and vaginal delivery of the unborn baby (“abdominovaginal approach”). We have not found a similar case in the literature of conjoined twin birth and we believe it is an experience worth sharing.

## 2. Case Presentation

A 35-year-old gravida 6, para 5 mother who is 38-week pregnant from last normal menstrual period has presented to Tercha General Hospital (a rural hospital in Southern Ethiopia). The patient is referred from a health center 60 kms far from this hospital for suspected “big baby” in labor. The patient was an illiterate housewife. In terms of past obstetrics history, all previous deliveries occurred at home vaginally with live birth with no major complication. During the index pregnancy, she had antenatal care visits at a nearby health center without ultrasound examination. She reports that the current pregnancy is heavier than previous ones and associated with significant discomfort than her previous pregnancy experiences. Otherwise, she has no self or family history of twinning in the past.

Examination shows a stable gravida with normal vital signs. Abdominal examination shows big for date uterus with two cephalic poles in the lower abdomen and positive fetal heartbeat. Standard ultrasound examination confirmed twin pregnancy with both in cephalic presentation and adequate amniotic fluid; single placenta with no visible dividing membrane; fetal heartbeat is visible at two sites and is in a normal range. Upon pelvic examination, the cervix is 8cm dilated with left occiput-anterior position at a station 0. Fetal membrane is ruptured with clear liquor passing. With diagnosis of twin pregnancy (both cephalic presenting), in active phase of first stage of labor patient is admitted to labor ward and management of labor started in the standard way.

**Figure 1:**
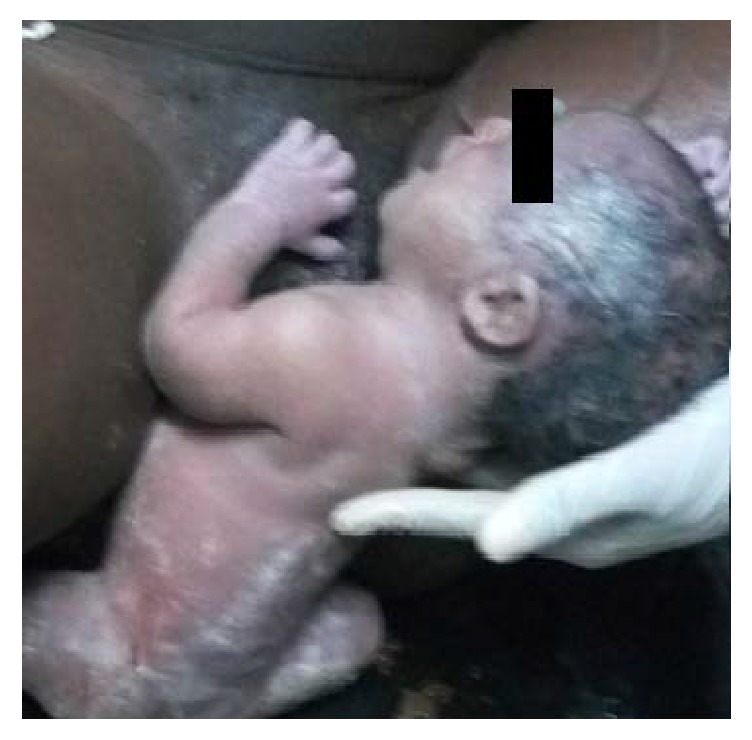
Baby 1 after delivery (after complete delivery of the body, the baby remained tightly attached to the perineum but is still crying).

In the next few hours labor progressed well and the first baby is crowning. Duty midwives are attending the delivery. Subsequently, with maternal effort the head and upper extremities of the first baby are delivered and the remaining part of the fetus is delivered by ‘gentle' traction by the midwives. But after delivery of the whole body, baby 1 remained ‘attached' to the mothers' perineum, though the baby is crying vigorously (Figure 1). The midwives started to shout for help and senior obstetrician arrived.

On reevaluation, we noticed the same and we found that the anterior abdomen of baby 1 from xiphisternum to the site of umbilical cord insertion is continuous into the uterine cavity. This led to sudden and unexpected consideration of the possibility of conjoined twins. Bedside ultrasound showed alive remaining fetus with fetal heart rate of 76 and in a transverse lie with the head in the right iliac fossa and fetal dorsum anterior. Initial attempt to access the extremities and aid delivery of the remaining fetus vaginally is not possible due to failure to reach the extremities for intrauterine manipulation. Emergency laparotomy is decided.

Emergency laparotomy under general anesthesia with midline subumbilical abdominal incision and lower uterine segment vertical hysterotomy is performed. We corrected the lie of the born baby such that it is parallel to that of the unborn baby; with deep vaginal examination along with the caudal end of the attachment and managed to manipulate the lower extremities of the intrauterine fetus to vagina and after grasping those with the right hand vaginally, we brought it to the perineum. Then there is careful manipulation to bring those extremities posterior to the born baby with a second assistant holding and manipulating the born baby away from the area of manipulation. Progressive delivery of the second baby of the conjoined pairs is affected by total breech extraction with minimal difficulty.

**Figure 2:**
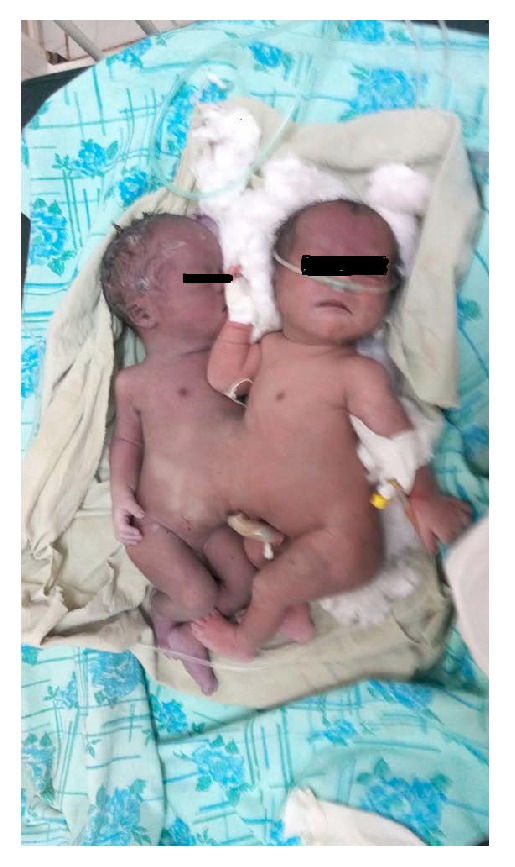
Omphalopagus twins delivered at Tercha General Hospital (the photo is taken at NICU after the death of the second born baby (left)).

Both newborns were depressed at completion of the procedure and recovered after aggressive resuscitation for 10 minutes (Figure 2). Both are male and their combined weight is 5800 gm. Ultrasound examination of the twins shows shared liver with no other organs shared. Latter the second baby passed away after 1 hour of stay at the NICU. The second baby died after 20 hours of stay, during transportation to higher center for possible emergency separation. The mother was discharged to home on her sixth post-op day after counseling.

## 3. Discussion

Conjoined twins represent one of the rarest forms of twin gestation. In this case report we present a unique and so far undescribed complication of a conjoined twin which is further complicated because of suboptimal antenatal care.

Though rare, the occurrence of conjoined twins has been described since 375 AD, where the birth of two-headed boy is reported in the castle of Emmaus to the original Siamese twins: Chang and Feng of Thailand [6]. In addition, it is not unusual to see/hear on the news about conjoined twins: controversies whether to separate, successful separation, and the like. In obstetrics practice also, there are several encounters with cases of conjoined twins, which lead to an extensive study on genesis, diagnosis, and outcome of these pregnancies. Although a lot has been said in this regard, a significant number of cases go undiagnosed until after birth, and obstetrical complications which can mainly be attributed to failure to timely diagnose have been reported in case reports. In resource-limited setting like Ethiopia, it is not unusual to see that obstetrical complications are detected for the first time after they developed a far serious stage and our case is not different [4–8].

In a setting where there is an optimal antenatal evaluation, diagnosis of conjoined twins can be made based on different clinical, ultrasound, and MRI criteria. When pregnancy is diagnosed; as part of a standard ultrasound examination, we need to determine the number of fetuses. And if a twin pregnancy is detected, chorionicity and amnionicity should be checked. Then in case of monochorionic, monoamniotic twin gestation, evolution is necessary to identify sharing of fetal organs. Monoamniocity, contiguous skin, twins that stay in the same orientation to one another, fetal scoliosis, unusual limb positioning, and more than three vessels in the cord are classical signs of conjoined twining [7]. However, the sonographic evaluation may not be reliable in case of oligohydramnios, obesity, and advanced pregnancy. In such cases, MRI may be helpful to diagnose as well as evaluate presence of other congenital anomalies as well as plan for possible separation surgery. In our patient, the fact that she had no antenatal ultrasound examination and the ultrasound examination performed intrapartum did not consider this possibility as the diagnosis is delayed.

When conjoined twin goes undiagnosed until term, several cases are reported where labor-related obstetrical complications occur and diagnosis is considered after occurrence of shoulder dystocia and obstructed labor reported [5–8]. When such complications occurred, different case specific interventions are reported in literature with different conditions associated with conjoined twins.

In the management of our case, we had four options.

### 3.1. Reposition of the Born Baby and Delivery by Caesarean Section

Mariatu B. et al. from Sierra Leone reported a neglected case of conjoined twins presented with obstructed labor after delivery of one head where delivery is finally achieved by caesarean section after repositioning of the born head [4]. However, in their case, only the head of the first baby is delivered. In our case, on the other hand, the whole body of the first baby is delivered and it is considered to be difficult to reposition the born baby. In addition, since both are alive, we believe that the extra time needed for reposition may contribute for an adverse perinatal outcome.

### 3.2. Destructive Delivery

In our hospital, we frequently perform destructive procedures to affect delivery of dead fetus with obstructed labor. One of these procedures, evesartion, is performed when we find cases of neglected, impacted shoulder presentation to double up and affect delivery. In a case report from Ethiopia, a successful caesarean delivery with destructive delivery (intraoperative decapitation) in a patient with conjoined twins presented for shoulder dystocia after ventouse delivery was reported [8].

However the fact that both babies are alive in this case prevented us from going for this procedure.

### 3.3. Vaginal Delivery of the Unborn Baby

This was the first procedure attempted in our case with no help. Probably, the site of the conjunction and the different presentation of our case made this possibility do not work for our case. We believe in smaller babies and after a destructive procedure this technique can be important

Maruti S. from India reported successful vaginal delivery of Dicephalus conjoined twin in breech presentation. While assisting the breach delivery the after coming head got stuck and failed to deliver. After liberal median episiotomy the fetus was delivered by traction and suprapubic pressure. The neonate is macerated weighing 2700gm with two heads [5].

### 3.4. Laparotomy, Hysterotomy, and Assisted Vaginal Delivery

The procedure we used to effect delivery in the current case is not used or reported in the literature of delivery of conjoined twins. Considering the tragedy that happened in our labor ward and the fact that the babies are alive, it has been an important measure that relived my team from the stress of seeing a crying baby die and witnessing a mother situation. In desperate situation like ours, it is invaluable to consider the most appropriate procedure that relives the complication. The fact that the babies died should not discourage us from taking the most appropriate action at hand.

Generally, the perinatal outcome of conjoined twins is poor. In one report, 28% of conjoined twins are stillbirths and up to 54% can end up in early neonatal death, with a survival rate of less than 8% [9]. Separation of those surviving twins can generally be considered electively at age 2 to 4 months of age. However emergency separation may be mandatory in case of death of one of the twins [10]. This is because of the fact that the death of one twin will inevitably lead to the death of the other unless an attempt is made to separate them because the surviving twin will exsanguinate into the dead twin. In our case, however, the surviving baby made for emergency separation during transfer to higher facility.

In conclusion, failure to diagnose conjoined twins during antepartum follow-up is common encounter in obstetric practices. The undiagnosed cases may present during labor for first time after they have caused serious complications. In such cases, technique used to complete delivery should be individualized based on clinical findings. Emergency separation is required in case of conjoined twins with the death of one twin.
